# Assessment of Startle Response and Its Prepulse Inhibition Using Posturography: Pilot Study

**DOI:** 10.1155/2016/8597185

**Published:** 2016-05-22

**Authors:** Jacek Polechoński, Grzegorz Juras, Kajetan Słomka, Janusz Błaszczyk, Andrzej Małecki, Agnieszka Nawrocka

**Affiliations:** ^1^The Jerzy Kukuczka Academy of Physical Education in Katowice, Katowice, Poland; ^2^Nencki Institute of Experimental Biology, Warsaw, Poland

## Abstract

*Purpose*. The aim of this study was to evaluate the possibility of using static posturography in the assessment of sensorimotor gating.* Subjects and Methods*. Fourteen subjects took part in the experiment. The inhibitory mechanisms of startle reflex were used as the measure of sensorimotor gating. It was evoked by a strong acoustic stimulus (106 dB SPL, 40 ms) which was preceded by the weaker similar signal (80 dB SPL, 20 ms). A stabilographic platform was used to measure sensorimotor gating.* Results*. Results of static posturography show that the postural sway caused by the reaction to a strong acoustic stimulus is significantly smaller when this stimulus is preceded by the signal of lower intensity (prepulse). Such assessment is only possible in eyes open conditions.* Conclusions*. Static posturography can be simple and effective method used for diagnosis of sensorimotor gating in humans.

## 1. Introduction

Sudden and strong acoustic signal causes orienting response prior to which a specific motor reaction can be observed. This response is called acoustic startle reflex (ASR) and can be decreased by prepulse inhibition (PPI), when a slight stimulus is previously generated. PPI is currently perceived as the measure of sensorimotor gating, being the ability of the nervous system to filter irrelevant information [1].

Assessment of sensorimotor gating is useful especially in neurological and psychiatric research. A lot of studies have shown that PPI is disturbed in many diseases and disorders, for example, schizophrenia [2–5], Alzheimer's disease [6], Multiple System Atrophy (MSA) [7], Parkinson's disease [8], generalized dystonia [9], and subcategory of autism, Asperger syndrome [10].

Startle response (startle reflex) is usually recorded using electromyography (EMG) and concerns the assessment of orbicularis oculi muscle activation [11–14]. It might be problematic, for example, in case of excessive blinking which can be an effect of nervous hyperactivity or motor tics. Therefore, the recording activity of and muscles (masseter, sternocleidomastoid, biceps brachii, abductor pollicis brevis) also is used in the assessment of startle reflex, [9, 15, 16]. Brown et al. [17] examined startle response caused by acoustic stimulus on leg muscles (tibialis anterior, soleus). However, such measurements are complicated and time-consuming. Experiments conducted on animals have shown that special force platforms can be used for assessment of startle response [18, 19].

The aim of this study was to evaluate the possibility of using the static posturography for assessment of the sensorimotor gating and startle reflex in human. The potential results of this study could be useful in diagnosis of patients with neuronal and psychological disorders.

## 2. Material and Methods

Studies have been approved by the Bioethical Commission of the Jerzy Kukuczka Academy of Physical Education in Katowice. Fourteen male students participated in the study (age 21.9 ± 1.1 years, height 182.2 ± 8.9 cm, and weight 78.6 ± 11.4 kg). The following exclusion criteria were specified: postural and auditory disorders and neurological or psychiatric deficits.

**Figure 1:**
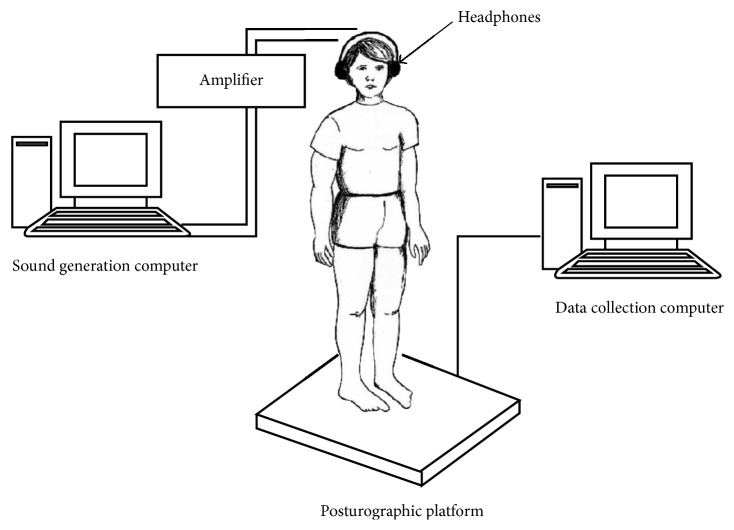
Experimental setup.

The centre of pressure (COP) sway in anterior-posterior (AP) and mediolateral (ML) directions was registered (at 40 Hz sampling frequency) using stabilographic platform (QFP/Medicapteurs, France) with Winposture 2000 software. Subjects were provided with acoustic pulses generated by Audacity software (release 1.2.4) and amplified by an external amplifier (UNITRA, model PW8010). The stimuli were delivered through headphones worn by each subject throughout the experiment (Figure 1).

**Figure 2:**
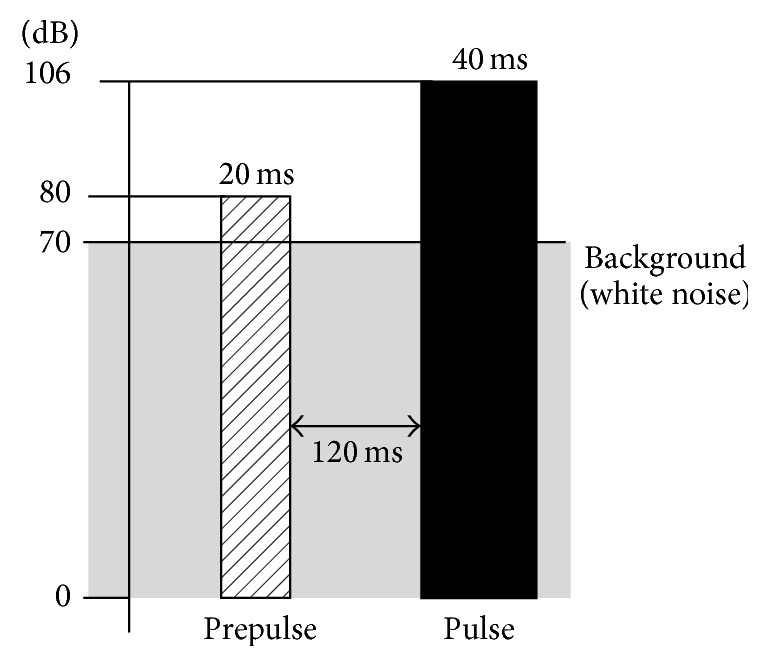
Time intervals and sound intensity of the acoustic stimuli.

Subjects were instructed to stand quietly on a stabilographic platform in a comfortable position with their arms along their sides. Each subject performed two trials with eyes open and two trials with eyes closed. The experimental procedure design was based on a similar study by Grillon et al. [20]. Startling stimuli (3x 106 dB, 40 ms) were presented alone or were preceded by prepulse stimuli (3x 80 dB, 20 ms) over a steady background noise (70 dB). The time between prepulse stimulus and startle stimulus was 120 ms (Figure 2). Analysis of posturographic parameters were conducted within 1.5 s after the startle stimuli were provided. Together six reactions to the stimulus and six reactions preceded by the prepulse stimulus were analyzed for each subject.

### 2.1. Statistical Analysis

Two-way analysis of variance (ANOVA) for repeated measures was used to determine the differences between responses to the different acoustic stimuli. The significance level was set at *p* < 0.05. All statistical analyses were performed using the Statistica software (9.0).

## 3. Results

The results of analysis in ML plane with eyes open show a significant effect of kind of stimulus on lateral sway, which was reduced when a sudden acoustic signal was preceded with prepulse stimulus (*p* < 0.005) (Table 1). In the rest of the trials there was no significant influence of stimulus kind (without Prepulse, with Prepulse) on the COP sway. Regardless of the plane and visual control, analysis of variance did not show a significant effect of the signals order on the amplitude of COP displacement (Table 1).

**Figure 3:**
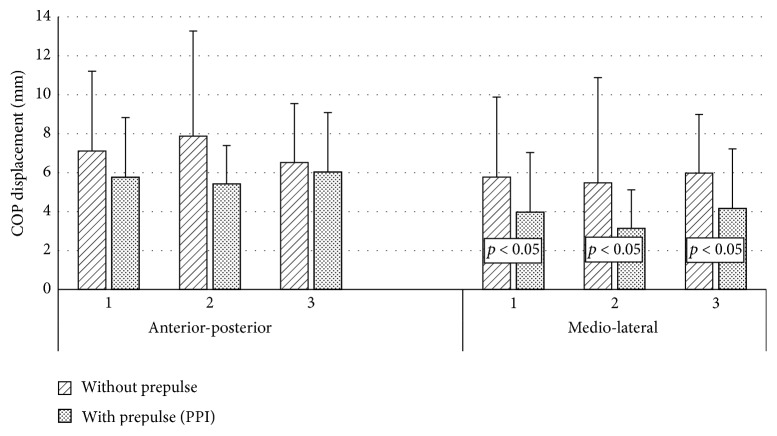
COP displacement depending on the kind (without Prepulse, with Prepulse) and order (1,2, 3) of the acoustic stimulus in eyes open trials.

In all consecutive acoustic stimuli with eyes open trial, weaker startle responses were observed when the signal was preceded with prepulse stimulus. However, the significant differences were found only in mediolateral plane. In three repetitions of acoustic stimuli, prepulse stimulus caused significant reduction of mediolateral COP displacement (Figure 3).

**Figure 4:**
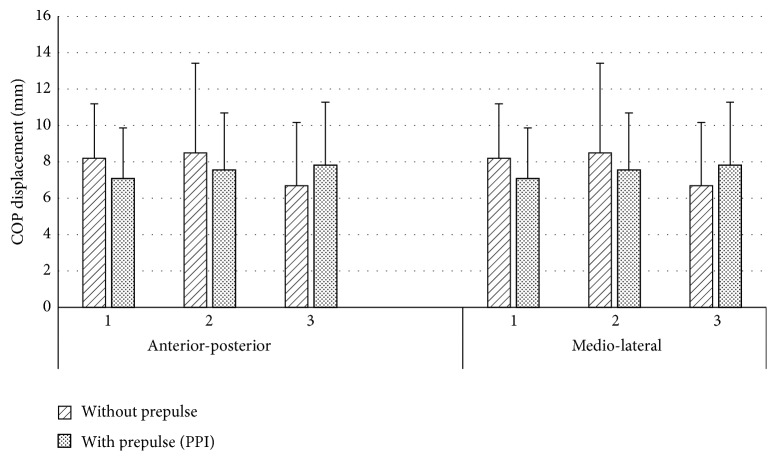
COP displacement depending on the kind (without Prepulse, with Prepulse) and order (1,2, 3) of the acoustic stimulus in eyes closed trials.

In all trials with eyes closed, the acoustic stimuli did not significantly influence the amplitude of COP displacement. Moreover, during the consecutive acoustic signals the weaker postural reactions were not always observed when the main stimulus was preceded by the prepulse (Figure 4).

**Figure 5:**
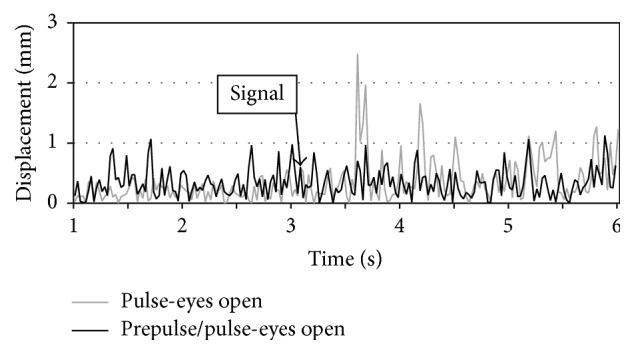
An exemplary stabilogram of COP displacement depicting the startle response caused by an unexpected acoustic signal (pulse) and its inhibition in case of prepulse signal.

The differences in reactions on startle and prepulse stimulus are shown on an exemplary stabilogram (Figure 5). A short time after the strong stimulus, the amplitude of COP displacement is significantly larger than in the case when the strong stimulus is preceded by prepulse.

## 4. Discussion

The results of this study show that unexpected acoustic stimulus of appropriate intensity elicits the startle response, which can be registered with the use of stabilographic platform. The presence of prepulse signal of a lower intensity causes the inhibition of postural reaction. Interestingly, such prepulse inhibition is only present in case of eyes open trials in ML plane.

The importance of visual information in the process of postural control is well documented, and together with the gravitational field it states extrinsic reference frame for the intrinsic mechanisms of postural control [21]. Previous studies concerning the process of postural control indicate its decline in case of lack of visual control [22–25]. It is possible that in trials with eyes closed, the startle response is masked by the higher indices of postural sway.

The statistically insignificant influence of prepulse on the inhibition of postural reaction in case of AP direction can be also caused by the masking effect of the startle response by the higher indices of postural sway in the sagittal plane. It is commonly known that postural sway in the sagittal plane is larger than in the frontal plane.

It seems that necessary condition to register prepulse inhibition with the use of posturographic platform is a strong startle response to the acoustic stimulus, characterized by the distinct postural reaction. Stronger reaction could be provoked by using higher intensity signals, exceeding 106 dB. Such sound profiles were used in other studies [26–28].

The magnitude of postural sway can be also determined by the initial position of subjects. Brown et al. [17] reported in their study that the startle response caused by acoustic stimulus is dependent on body position and muscle activation. Authors registered different EMG profiles of leg muscles (tibialis anterior, soleus) in standing and sitting position in response to an acoustic stimulus.

Startle response observed with the use of force platform, as an effect of acoustic stimuli, was also examined by Hillman et al. [29]. They indicated a positive correlation of blinking reflex and whole body startle. Stronger blinking reflex correlated with a stronger general response. This relationship was observed only in the sagittal plane. According to these authors a distinct postural reaction caused by an unexpected acoustic stimulus can elicit general flexors activation. In our research stronger acoustic startle response was also noticed in the AP plane, as indicated by the greater COP displacement. However, significant prepulse inhibition was found only in the frontal plane (mediolateral). It may be another indication that excessive COP displacement impede the assessment of prepulse inhibition (masking effect).

The dependency between whole body startle response and blinking in the presence of unexpected stimulus reported by Hillman et al. [29] as well as our previous studies indicates that the static posturography methods can be an alternative to the electromyography of muscles during the assessment of sensorimotor gating and diagnosis of certain psychic and neurological disorders. It should be noticed that startle reflex is generalized reaction which includes multiple muscle group at the same time, and therefore it seems necessary to its global assessment, which is possible using posturography. Whole body startle response examination can be also very important in sports activities especially in case of the reactions to the start signals. However, the use of static posturography in neurological and sports diagnosis needs further experiments in order to improve the procedure.

*Implication for Further Studies*. Our study provides new approach of the assessment of startle response and its prepulse inhibition using posturography. Further studies are needed to improve and standardize this type of procedures (that includes a large sample size, both genders, and, above all, synchronous measurement of electromyography and posturography).

## 5. Conclusions

1. Static posturography allows registration of postural sway due to startle response effects in humans.
2. The significant weakening of the startle reaction in the presence of prepulse stimulus is observed in mediolateral plane with eyes open.
3. Static posturography can be used as a simple effective method in the assessment of sensorimotor gating in humans. It is necessary to improve and standardize the testing procedures.

## Figures and Tables

**Table 1:** The influence of the kind (without prepulse, with prepulse) and order of the acoustic stimuli on the COP displacement.

Variables	Eyes open				Eyes closed			
	Anterior-posterior		Mediolateral		Anterior-posterior		Mediolateral	
	*F*	*p*	*F*	*p*	*F*	*p*	*F*	*p*
Stimulus kind	2.352	0.149	14.102	**0.002**	0.179	0.679	0.007	0.935
Stimulus order	0.130	0.879	0.604	0.554	0.435	0.652	0.328	0.724
Stimulus kind × stimulus order	0.875	0.429	0.146	0.865	0.948	0.400	0.244	0.785
